# Supplementary figures and images for: A longitudinal characterization of sex-specific somatosensory and spatial memory deficits in HIV Tg26 heterozygous mice
Source: PLoS One. 2020 Dec 31;15(12):e0244725. doi: 10.1371/journal.pone.0244725 (PMC7775086; doi:10.1371/journal.pone.0244725)

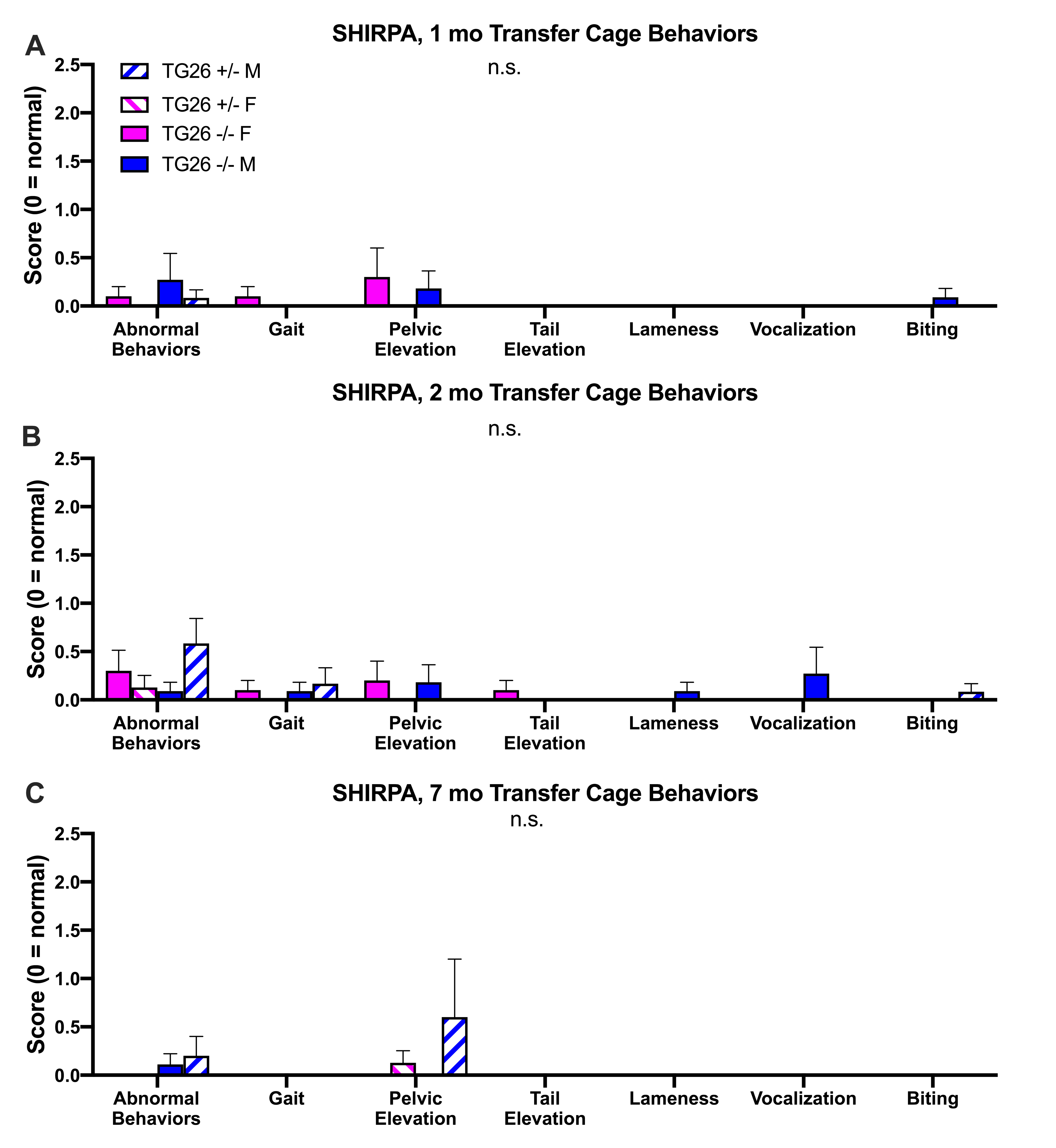

Supplement: S1 Fig — Remaining transfer cage behaviors from general health screen testing, in wild-type mice -/- and heterozygous Tg26 +/- mice of both sexes, across time (1, 2 and 7 months of age). No significant differences (n.s.) were observed between groups for these behaviors. (TIF) [file pone.0244725.s001.tif]

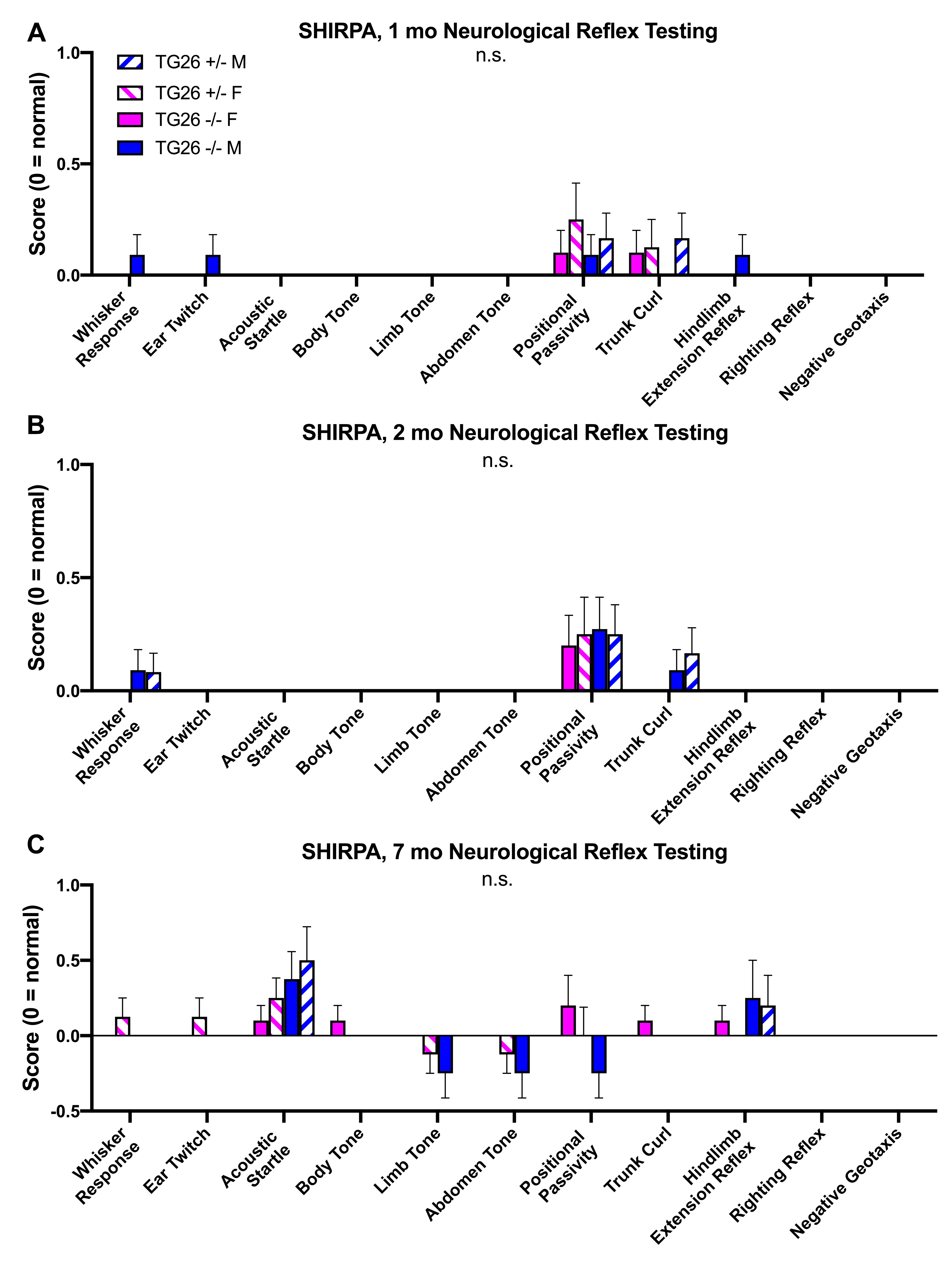

Supplement: S2 Fig — Remaining general neurological health screenings results that were not shown in Fig 2, in wild-type mice -/- and heterozygous Tg26 +/- mice of both sexes, across time (1, 2 and 7 months of age). No significant differences (n.s.) were observed between groups for these behaviors. (TIF) [file pone.0244725.s002.tif]
